# Supplementary material for: An interpretable framework for clustering single-cell RNA-Seq datasets
Source: BMC Bioinformatics. 2018 Mar 9;19:93. doi: 10.1186/s12859-018-2092-7 (PMC5845381; doi:10.1186/s12859-018-2092-7)
Supplement: Supplementary file 1 — Supplementary figures and separation score analysis. A file containing a distance-metric interpretation of separability score along with the three supplementary figures described in the main text: 1) visual analysis of the Yan et al. dataset, 2) further analysis for the splits generated for the Zheng et al. dataset, 3) analysis on the full Zheng et al. dataset. (PDF 3707 kb) [file 12859_2018_2092_MOESM1_ESM.pdf]

Supplementary material

Separation score interpreted in terms of a distance metric

The separation scored used for all experiments described in the main text is defined as

$$s(\mathbf{X},\mathbf{Y}) = -\log_{10}(\min_i p(\mathbf{x}_i,\mathbf{y}_i))$$

and quantifies how different the $N_1 \times M$ population $\mathbf{X}$ is from the $N_2 \times M$ population $\mathbf{Y}$. For notational simplicity, we let $\mathbf{X} = [\mathbf{x}_1 \ \dots \ \mathbf{x}_M]$ where $\mathbf{x}_i$ is the N_1-dimensional vector of counts for gene i in population $\mathbf{X}$. We use similar notation for $\mathbf{Y}$. $p(\mathbf{x}_i,\mathbf{y}_i)$ represents the p-value achieved using some differential expression test for gene i. Although we used Welch's t-test in the main text (unequal variances, $N_1 \neq N_2$), we can gain some insight for this separation score if we consider what would the score would look like with the Student's t-test (equal variances, $N_1 \neq N_2$). For gene i, a Student's t-test starts by computing the t-statistic:

$$t_i = \frac{\bar{x}_i - \bar{y}_i}{s_i \cdot \sqrt{\frac{1}{N_1} + \frac{1}{N_2}}}$$

where $\bar{x}_i$, $\bar{y}_i$ are the sample means of the gene in each population, and s_i is an estimator for the pooled standard deviation for the gene across both populations. To compute a p-value, we compute the area under a t-distribution with $N_1 + N_2 - 2$ degrees of freedom above $|t_i|$. This area can only decrease as $|t_i|$ increases, and hence $p(\mathbf{x}_i,\mathbf{y}_i) = f(\frac{1}{s_i}|\bar{x}_i - \bar{y}_i|)$ where f is a monotonically decreasing function. Furthermore, because N_1 and N_2 are constant across genes, the t-distribution and consequentially f are identical across genes.

We can interpret this score using a distance metric between $\mathbf{X}$ and $\mathbf{Y}$ after each population is mapped to a M-dimensional feature vector ϕ:

$$\phi(\mathbf{X}) = \begin{bmatrix} \bar{x}_1/s_1 \\ \bar{x}_2/s_2 \\ \vdots \\ \bar{x}_M/s_M \end{bmatrix}, \qquad \phi(\mathbf{Y}) = \begin{bmatrix} \bar{y}_1/s_1 \\ \bar{y}_2/s_2 \\ \vdots \\ \bar{y}_M/s_M \end{bmatrix}.$$

We see that the separation score can be rewritten as

$$\begin{aligned} s(\mathbf{X},\mathbf{Y}) &= -\log \min_i p(\mathbf{x}_i,\mathbf{y}_i) \\ &= -\log \min_i f\left(\frac{1}{s_i}|\bar{x}_i - \bar{y}_i|\right) \\ &= -\log f\left(\max_i \frac{1}{s_i}|\bar{x}_i - \bar{y}_i|\right) \\ &= -\log f(\|\phi(\mathbf{X}) - \phi(\mathbf{Y})\|_\infty). \end{aligned}$$

We see now that, for the Student's t-test, the separation score is a monotonically increasing function of the L_∞ distance (or the Chebyshev distance) between the feature vectors $\phi(\mathbf{X})$ and $\phi(\mathbf{Y})$.

References

1. Yan, L. *et al.* Single-cell rna-seq profiling of human preimplantation embryos and embryonic stem cells. *Nat Struct Mol Biol* **20**, 1131–1139 (2013). URL http://dx.doi.org/10.1038/nsmb.2660.

2. Zheng, G. X. Y. *et al.* Massively parallel digital transcriptional profiling of single cells. *Nature Communications* **8**, 14049 EP – (2017). URL http://dx.doi.org/10.1038/ncomms14049.

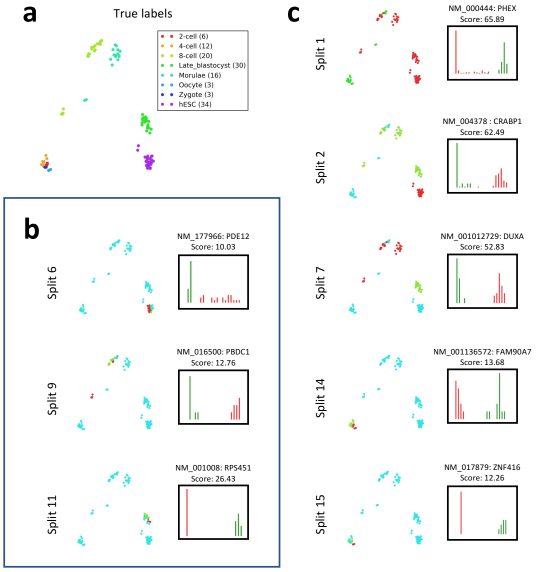

Supplementary Figure 1. Exploratory analysis on Yan et al. dataset. During analysis of the 124 cells in the[1] dataset (Figure 3 in the main text), DendroSplit generated labels after the split step that disagreed with the ground truth labels. **(a)** The ground truth labels provided by[1] are shown. **(b)** We see that some cell types, such as the hESCs, were oversplit. **(c)** Other splits were correct, yielding clusters that are consistent with true labels.

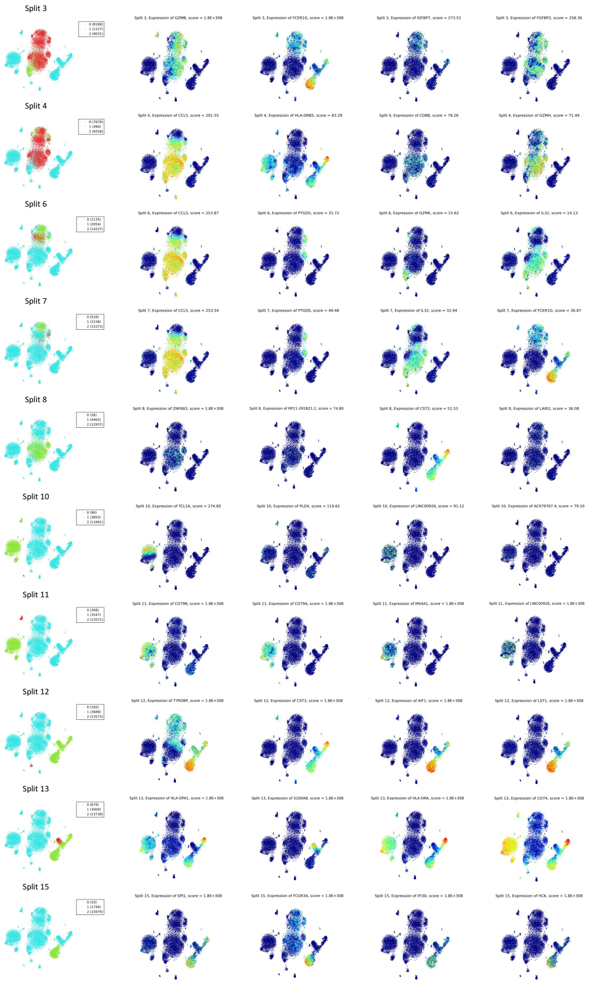

Supplementary Figure 2. Exploratory analysis on PBMC dataset continued. This Figure supplements Figure 6 in the main text, providing exploratory analysis for the splits not visualized in Figure 6B. The dataset consists of 17426 cells, 908 features (genes) from fresh peripheral blood mononuclear cells (PBMCs)[2].

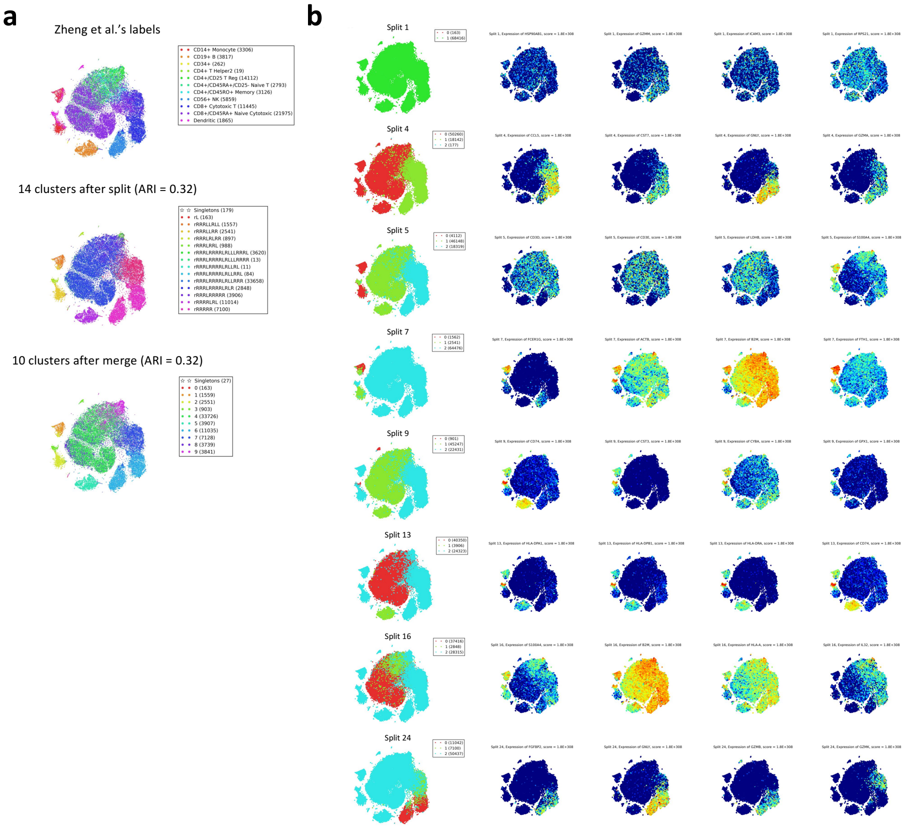

Supplementary Figure 3. Exploratory analysis on unfiltered PBMC dataset. The experiment visualized in Figure 6 in the main text is repeated without any filtering of genes and cells. The dataset consists of 68579 cells, 20374 features (genes) from fresh peripheral blood mononuclear cells (PBMCs)[2]. Gene expression is quantified using UMI counts. **(a)** The labels generated by DendroSplit after the split and merge steps are visualized using a tSNE embedding of the data points. The split and merge thresholds are 400 and 200, respectively. Generating the distance matrix takes 2672.96 seconds, and performing the split and merge steps takes 7059.34 seconds. **(b)** 9 of the 26 recorded valid splits are shown along with the expression levels of the top 4 genes used for validating each split.
